# Exercising With a Six Pack in Virtual Reality: Examining the Proteus Effect of Avatar Body Shape and Sex on Self-Efficacy for Core-Muscle Exercise, Self-Concept of Body Shape, and Actual Physical Activity

**DOI:** 10.3389/fpsyg.2021.693543

**Published:** 2021-10-08

**Authors:** Jih-Hsuan Tammy Lin, Dai-Yun Wu, Ji-Wei Yang

**Affiliations:** ^1^Department of Advertising, College of Communication, National Chengchi University, Taipei City, Taiwan; ^2^Taiwan Institute for Governance and Communication Research, Taipei City, Taiwan; ^3^Department of Communication and Technology, National Yang Ming Chiao Tung University, Zhubei, Taiwan

**Keywords:** avatar body shape, self-concept, self-efficacy, female and male, Proteus effect, virtual reality, virtual reality exercise, physical activity

## Abstract

This study investigates the Proteus effect from the first-person perspective and during avatar embodiment in actual exercise. In addition to the immediate measurements of the Proteus effect, prolonged effects such as next-day perception and exercise-related outcomes are also explored. We theorized the Proteus effect as altered perceived self-concept and explored the association between virtual reality (VR) avatar manipulation and self-concept in the exercise context. While existing studies have mainly investigated the Proteus effect in a non-VR environment or after VR embodiment, we aim to contribute to the literature by addressing this concern to explore how the Proteus effect works in actual VR exercise. Through a 2 (avatar body shape: with a six pack vs. normal) × 2 (sex: male vs. female) between-subject experiment, the results partially support the Proteus effect. Regarding actual physical activity, embodying an avatar with a six pack during exercise creates fewer body movements. No significant effect was found for perceived exertion. We also explored the role of sex as a potential moderator in the association of the Proteus effect on exercise outcomes. The Proteus effect was supported by immediate and next-day self-efficacy for core-muscle exercise only among female participants. The between-subject design allowed us to probe how avatar manipulation of muscular body shape with a six pack as opposed to normal body shape influences participants’ self-concept and exercise outcomes, as limited VR studies have employed within-subject comparisons. This also contributes to the literature by providing an upward comparison (e.g., muscular with a six pack vs. normal) as opposed to the previous downward comparison regarding body fitness (e.g., normal vs. obese). The overall results supported the Proteus effect in the context of core-muscle exercise when comparing normal and ideal body shape avatars. However, the Proteus effect as an altered self-concept and its effects on self-efficacy for exercise were supported among females but not males. Whereas the female participants who embodied avatars with a six pack associated themselves more with the muscular concept than other people, the male participants who embodied avatars with a six pack perceived themselves as more normal than others. Theoretical and practical implications are discussed.

## Introduction

Embodiment in virtual reality (VR), also termed the body ownership illusion (BOI) (Slater, 2009), is the illusion by which healthy people perceive an avatar’s virtual body as if it were their own physical body even though they know it cannot be (Maselli and Slater, 2013). Unlike other media, in which audiences view the avatar from the third-person view or through the computer screen, embodiment in VR allows one to perceive the virtual body as his/her own. Several empirical examinations of virtual body illusions have indicated that one demonstrates attitudes or behaviors that are consistent with the traits of virtual avatars during and after virtual avatar embodiment, termed the Proteus effect (Yee and Bailenson, 2007; Yee et al., 2009). Although not every relevant study adopts the term Proteus effect, research on the effects of embodiment has examined the basic concept: how do users behave and what is their attitude after embodying a virtual avatar with assigned traits?

Although the original study that coined the term Proteus effect tested this effect in VR, subsequent studies have examined this effect in digital games and console games that present avatars from a first-person or third-person view on the screen. The results are inconsistent, with some supporting the effect (Peña et al., 2009) and others unable to replicate it (Sylvia et al., 2014). In this study, we discuss the Proteus effect in VR only from a first-person perspective, which provides the full BOI and its effects. Among published VR studies, most have confirmed the Proteus effects. For example, a 40-year-old woman who embodied the virtual body of a 4-year-old girl later overestimated object size (Banakou et al., 2013), supporting the Proteus effect. Users who embody a coral (Ahn et al., 2016) experiencing ocean acidification perceive themselves to be closer to nature and thus are more motivated to pay attention to environmental issues.

With VR equipment becoming more accessible and easier to use, for example via standalone VR goggles, using VR to exercise has become a trend, especially during the COVID-19 pandemic (Hart, 2021; Siani and Marley, 2021). Existing applications provide various content for users to engage in boxing, dancing, or aerobic exercises, but none of these incorporate virtual body elements. What effect is had by exercising with a six pack? Will we be more motivated to exercise and perceive ourselves to have strong core muscles? No research has examined the Proteus effect in the context of VR exercise from a first-person perspective, as existing studies mostly project the avatar appearance on a mirror in the VR environment (Kocur et al., 2020) or present the avatar from the third-person view (Fox and Bailenson, 2009; Bailenson and Segovia, 2010). Examining the Proteus effect in the context of VR exercise from the embodiment perspective can shed light on the literature and practical design.

In the current study, we examined the Proteus effect through avatar manipulation of body shapes in VR, including a body with six packs and a normal-shaped body, among male and female participants in the context of promoting core-muscle exercise. We also examined the automatic self-concept as the theoretical underlying mechanism of the Proteus effect.

### Avatar Manipulation in an Exercise Context

Using communication technology to promote exercise efficacy and behavior has consistently received scholarly attention. Existing studies testing avatar manipulation in the context of exercise have mostly employed digital games such as those available on the Nintendo Wii and other video game interfaces (Peña et al., 2016; Navarro et al., 2020). Although the avatar manipulation was not conducted in VR, these studies suggested that they were testing the Proteus effect. Most of these studies manipulated avatars’ body shapes, such as normal versus obese body shape, and examined the effect on exercise intention and physical activity. For example, Joo and Kim (2017) assigned participants to 2 (obese vs. normal avatar body shape) × 2 (healthy vs. unhealthy lifestyle) groups in Sims 4 to examine how these avatar traits affected the participants’ exercise (i.e., stepping machine) and actual cookie consumption after the virtual game experience. The results indicated that neither the avatar shape nor the lifestyle affected the participants’ exercise behaviors or unhealthy eating behaviors. However, they found an interaction effect on exercise behavior. Among the participants playing Sims 4 with a normal-weight avatar, those who had a healthy lifestyle avatar engaged in more steps on the stepping machine than those with an unhealthy lifestyle avatar. They suggested that the participants did not want to learn from obese avatars and that avatars with an ideal body shape and positive lifestyle influenced the participants’ exercise behaviors. The Proteus effect was not significant but was effective in certain conditions.

In another study (Li et al., 2014), overweight children aged 9–12 years old were assigned to 2 (avatar shape: normal vs. overweight) × 2 (stereotype threat messages: present vs. absent) conditions to play Wii games. The results supported the Proteus effect in that overweight children playing the game through normal body size avatars had greater exercise attitudes, general exercise motivation, exercise motivation using Wii, and game performance than those assigned avatars with an overweight body shape. A conditional Proteus effect was identified in which overweight children with a stereotype threat absent condition reported greater exercise attitude and motivation than those with an avatar of normal body shape. This indicated that the Proteus effect can have different effects depending on the context and message frame.

The Proteus effect also occurs in the downward comparison condition in exercise when an opponent embodies a less ideal body than the participant in a competition requiring physical activity. Scholars (Peña et al., 2016) examined how avatar body size and opponent body size affected male participants’ physical activity during a competitive exergame. They found a main effect of the Proteus effect: male participants operating normal body size avatars demonstrated greater physical activity than those operating obese avatars. They also found an interaction effect in which participants demonstrated decreased physical activity when the opponent avatar’s body was perceived as more obese than their own avatar’s. In addition, if the participants perceived their own avatar to be more obese than their opponent’s, they showed decreased physical activity. These results showed that the social comparison of body size influenced the participants’ physical activity through different processes. The study did not measure the underlying mechanisms, which may explain such moderated differences in physical activity.

In another study, Navarro et al. (2020) examined whether avatar clothes (sports or formal clothes) and faces (self or strangers’ faces) affected participants’ physical activity. The results showed an interaction effect in that the participants operating avatars with their own faces and with sport clothes demonstrated greater physical activity than those in the other conditions. In addition, those who operated avatars with a stranger’s face and formal clothes demonstrated decreased physical activity. This indicated that even the clothes on the avatar affected the participants’ physical activity.

### Proteus Effect in Virtual Reality

Since the Proteus effect was first identified in virtual reality (Yee and Bailenson, 2007), scholars have interpreted Proteus effects through various approaches, such as self-perception (Yee and Bailenson, 2007), priming (Peña et al., 2009), or schema activation (Ratan and Dawson, 2016). Despite the various interpretations, scholars commonly agree that the Proteus effect is a top-down avatar induced “attitudinal and behavioral change through self-perception” among users (Ratan et al., 2020, p. 654). Empirical evidence indicates that when users embody an avatar with characteristics different from their own, they hold attitudes and exhibit behaviors consistent with the traits of the avatars. For example, individuals who embody a taller avatar are more willing to turn down unfair offers than those who embody a shorter avatar (Yee and Bailenson, 2007), indicating that embodying taller avatars enables participants to be more confident in their decisions.

A meta-analysis (Ratan et al., 2020) shows that the Proteus effect through avatars is larger than other media effects, such as the aggression or prosocial effects of video games, found by studies that focus on the effect of media content on audiences. This indicates that persuasive characteristics through embodied avatars exert a stronger influence on users than other types of media since users interact with digital content through the representation of the avatar. The inconsistent results regarding the Proteus effect in the literature may be due to avatar induction being accomplished through multiple approaches, including digital games, console games, or VR. The distinctive difference between VR embodiments and others lies in individuals being able to look down at their own virtual body as if it were their own. This first-person no-distance view provides a strong BOI, which results in greater effects than other modes (Yee and Bailenson, 2009; Ahn et al., 2016). Therefore, we focused on the Proteus effect in VR in the current study.

Among VR studies examining the Proteus effect, most studies have confirmed the Proteus effect. Banakou and colleagues examined avatar manipulation in various contexts and found that individuals change their attitude and behavior and conform to the traits of embodied avatars. For example, Caucasian participants who embodied an African American dark-skinned avatar demonstrated a significant reduction in racial bias (i.e., viewing themselves as closer to an African American outgroup) according to the Implicit Association Test (IAT; Banakou et al., 2016) compared to Caucasian participants who embodied a white-skin avatar or purple-skin avatar (as a control group). In another study, 40-year-old women who embodied 4-year-old girl avatars subsequently spoke at a higher frequency, which indicates conforming to the child’s high frequency speech (Tajadura-Jiménez et al., 2017). Seinfeld et al. (2018) invited participants with a history of domestic violence to embody female victim avatars to experience abuse. They found that this embodiment allowed these male participants to experience physical and verbal abuse first hand in VR, and these participants reported a greater reduction in bias to recognize fear emotions than those in the control groups. Although the above scholars never used the term Proteus effect in any of their research, the work regarding virtual embodiment provides ample evidence to support the Proteus effect (Yee and Bailenson, 2007): the top-down avatar traits affected individuals’ attitudes and behaviors and made them consistent with the assigned traits through embodiment.

In addition to manipulating individuals’ traits through virtual avatars, research has also examined embodying individuals in non-human organisms. Ahn et al. (2016) invited participants to embody a coral to see the coral’s arms (as if they were the participants’ arms) falling due to ocean acidification. Participants who embodied a coral in VR experienced greater inclusion of nature in the self through embodiment than those who watched a video, and the inclusion of nature in the self further led to greater issue involvement through a decrease in perceived temporal distance. In another experiment (Ahn et al., 2016), participants embodying a cow in VR had greater spatial presence and embodiment than those who watched a video, and embodiment mediated the effects of VR on the inclusion of nature in the self. These results indicated that VR embodiment has greater effects than video embodiment, and such effects are not limited to human traits but extend to non-human living organisms through the perception of the inclusion of others in the self.

### Proteus Effect in Virtual Reality Exercise

Most existing studies examining the Proteus effect in VR have focused on the context of social interaction or issue persuasion (Ahn et al., 2016; Banakou et al., 2016), and research has only recently employed VR embodiment in the context of exercise (Kocur et al., 2020). With the increasing popularity and affordability of VR equipment via standalone machines, users can easily use VR goggles and engage in exercise. This is especially useful in the context of pandemics, such as COVID-19, that require social and physical distancing. Existing research (Peña et al., 2016) has shown that avatar manipulation affects players’ exercise outcomes, and we hypothesize that the Proteus effect could increase users’ exercise motivation through avatar manipulation in VR. Specifically, VR enables the BOI among users in which they perceive the avatar’s body as their own because they can look down at it as their own (Slater, 2009).

Two studies have examined Proteus effects during VR exercise. Reinhard et al. (2020) examined whether the age of participants’ avatars (old versus young) affected their walking speed in VR, and they found that the Proteus effect diminished quickly. The participants who previously embodied an older avatar required more time to walk the same set distance after the embodiment than those who previously embodied younger avatars and those in the control group (i.e., non-VR group). However, this effect occurred only in the first session after the stimulus and not the second section. In the second session, all three groups displayed similar times. This could be because the study tested walking behavior *after* embodiment with virtual avatars rather than testing walking speed *during* embodiment. Therefore, the after-stimulus effect diminished quickly.

The second study (Kocur et al., 2020) examined the Proteus effect during VR avatar embodiment. In this exploratory study with 30 participants (15 male and 15 female) and a within-subject design, the authors examined the effect of avatar muscularity (three levels: non-muscular, medium, and muscular body) on the participants’ physical performance and perceived exertion. They found a Proteus effect of the avatar’s muscular appearance on the participants’ perceived exertion, in which the participants embodying muscular avatars reported a lower perceived exertion (i.e., perceived the task to not be that hard) than when they embodied non-muscular avatars. In addition, they found a Proteus effect of the avatar’s muscular appearance on the participants’ grip strength, but this effect occurred only among male participants, indicating sex to be a moderator. They suggested that the Proteus effect in the context of a muscular avatar appearance can decrease participants’ perceived effort and increase grip strength. This study provided an initial examination of the Proteus effect *during* avatar embodiment. However, the participants performed short physical strength tasks, such as lifting a physical weight and putting it back, rather than actual exercise. It is unclear how and how long Proteus effects work during avatar embodiment in actual exercise sessions. In addition, the within-subject design in which each of the 15 participants embodied each of the three avatar types and reported all perceptions repetitively could introduce bias and increase type II errors. More research on the Proteus effect during and after avatar embodiment in an actual exercise context using a between subject experimental design is needed.

### The Outcomes of the Proteus Effect in Virtual Reality Exercise

There are important physical and psychological outcomes of exercise. Research regarding Proteus effects on exercise focus on physical outcomes including the physical activity (Reinhard et al., 2020) and perceived exertion (Kocur et al., 2020). Physical activity is the actual movement one engages during exercise, and can be objectively measured by an accelerometer or subjectively reported by participants, such as by using the IPAQ questionnaire (Dyrstad et al., 2014). Accelerometer-measured physical activity captures the objective physical movements one engages in through measuring the vector magnitude of movements in three axes (Dyrstad et al., 2014): the *X*-axis representing horizontal dimension when the participant moves right and left, the *Y*-axis reflecting up and down movements, and the *Z*-axis representing back and forth movements. Counts of physical movements determine one’s intensity of physical activity in a certain frame (Peng et al., 2015), and can reflect one’s lifestyle considering the sedentary time in a longer period of time (Troiano et al., 2008; Peng et al., 2015). In the current experiment, we focus on the actual body movements in a short period of time to reflect physical activity. Perceived exertion is the perceived level of effort committed to an exercise and estimates the “effort and exertion, breathlessness, and fatigue during physical work” (Borg, 1998, p. v; Williams, 2017, p. 404). Perceived exertion is usually measured by the self-reported Borg rating of perceived exertion. Accelerometer data provide an objective report of one’s actual physical activity, whereas perceived exertion reflects one’s perceived effort devoted to the exercise (Borg, 1998; Peng et al., 2015).

The psychological outcomes of exercise center on the motivation to exercise regularly (McAuley and Blissmer, 2000; Thøgersen-Ntoumani and Ntoumanis, 2006; Cataldo et al., 2013). Based on a core construct in social cognitive theory, self-efficacy is defined as “the belief in one’s capabilities to organize and execute the courses of action required to produce given attainments” (Bandura, 1997, p. 3). In the context of exercise, exercise self-efficacy is the belief that one is capable of exercising and is willing to perform the assigned action (i.e., exercise) in the future. Exercise self-efficacy is an important predictor of the adoption and maintenance of exercise behaviors (Fletcher and Banasik, 2001). High exercise self-efficacy leads to higher initiation and continuation of exercise behaviors.

In this study, we aimed to investigate the Proteus effect from the first-person perspective and during avatar embodiment in actual exercise. In addition, next-day perception and exercise-related outcomes were also explored. We chose the context of core-muscle exercise and examined how an avatar’s muscularity (cues that elicit the Proteus effect) affects participant exercise outcomes. In the context of engaging in core-muscle exercise, if one has muscular core muscles, one has the core power to maintain balance and limit extraneous movements around when performing related exercises. For example, when one performs squats, which exercises core and other muscles, if one has strong core muscles, one can stably perform such a task with fewer extraneous body movements than those with weak core muscles. Therefore, muscular participants require only certain physical motions to complete the exercise. Similarly, muscular participants should perceive lower exertion when performing related exercises (Kocur et al., 2020). Therefore, we proposed the following hypotheses based on the Proteus effect. We refer to normal-body avatars as avatars without core muscles and avatars with obvious core muscles as muscular-body avatars.

**H1:** Participants embodying normal-body avatars will engage in greater physical activity than participants embodying muscular-body avatars.

**H2:** Participants embodying normal-body avatars will report greater perceived exertion than participants embodying muscular-body avatars.

Other than the physical outcomes, how does the Proteus effect during VR exercise affect psychological reactions to the exercise? Does embodying an avatar with an ideal body image increase or decrease the sense of self-efficacy for exercise? According to the existing literature, body image may be either positively or negatively associated with exercise efficacy and motivation. Ouyang et al. (2020) indicated that positive body image can enhance self-efficacy for exercise and increase sports participation. In another study, Song et al. (2011) found that seeing the unsatisfactory self-image on a screen while playing the exergame may decrease exercise self-efficacy because the self-image reminds oneself of the discrepancies between self and the ideal image. However, plenty of studies found that negative body image or the intention to improve the self-image (i.e., trying to appear toned or fit) motivates people to exercise more (Conroy et al., 2000; Hausenblas et al., 2004; Brudzynski and Ebben, 2010). Based on the inconsistent evidence in the literature, we developed two competing hypotheses:

H3-1: Participants embodying muscular-body avatars will report greater (a) immediate self-efficacy and (b) next-day self-efficacy for core-muscle exercise than participants embodying normal-body avatars.

H3-2: Participants embodying normal-body avatars will report greater (a) immediate self-efficacy and (b) next-day self-efficacy for core-muscle exercise than participants embodying muscular-body avatars.

### Proteus Effect as an Altered Automatic Self-Concept

Scholars have theorized associations between avatars and players in digital games (i.e., character identification, Klimmt et al., 2009) and in VR (i.e., Proteus effect, Yee and Bailenson, 2007). Several theories have been employed to explain the Proteus effect. Yee and Bailenson suggested that in VR, avatar traits are limited cues associated with the self, so the first-person perspective embodiment changes one’s self-perception. Other scholars have suggested the Proteus effect to be due to a priming mechanism (Peña et al., 2009) or schema-activation mechanism (Ratan and Dawson, 2016), and both stressed that individuals form their self-concept based on the limited cues from the embodied avatar.

Regardless of the underlying mechanism, the above theoretical arguments center on the result that players form an altered perceived self-concept based on the assigned avatar cues and traits. Existing empirical evidence has implicitly provided support for the argument that VR embodiment produces an altered self-concept. Tajadura-Jiménez et al. (2017) found that older women who embodied a 4-year-old girl VR avatar reported a younger perceived self-concept than those who embodied a 60-year-old VR avatar. In another experiment (Hasler et al., 2017), white participants embodied a black VR avatar and treated black people as a novel in-group and thus reported decreased racial bias toward black people compared with those who embodied white VR avatars. Although Slater and his colleagues did not use the term Proteus effect in any of their studies, their results show that virtual body ownership results in self-concept changes. Therefore, here, we argue that the Proteus effect is due to an altered automatically perceived self-concept. We thus propose the following hypotheses:

H4: Participants embodying muscular-body avatars will perceive their own body as being more muscular than participants embodying normal-body avatars.

### Sex as a Moderator

Previous literature has suggested that responses toward idealized body shape and body image vary with sex, with women being more inclined than males to internalize an idealized body image and more likely to experience body-related schema activation from viewing thin-ideal images (Hargreaves and Tiggemann, 2003). The literature also indicates that women view thin as well as thin and muscular (i.e., toned) bodies but not hypermuscular bodies as ideal (Benton and Karazsia, 2015). Sex is a potential moderator of the Proteus effect on altered self-concept and should be explored.

The above concept of ideal body is derived from the Western standard. In the Taiwanese context, weight training has become popular in recent years and having a six pack with a thin body has been glorified as an ideal body shape by several women celebrities (Song and Huang, 2020; Ciao, 2021). Weight training for core-muscle exercise is also emphasized among society, and university students adopt this as their exercise routine (Taiwan Sports Administration, 2020; Taiwan Health Promotion Administration, n.d.; Zhou, n.d.). The consensus in Taiwanese society is that having a six-pack core is an ideal body condition regardless of sex. Although ideal body shape depends on individual preferences, core-muscle training to form a six pack is equally empathized for both sexes in Taiwan. Therefore, in this study, we explore potential sex difference in the Proteus effect when participants embody an avatar with a six pack.

In addition to different responses toward the level of muscularity, male and female participants also report different patterns toward exercise and exercise self-efficacy. Female participants in exercise normal interventions report increased aerobic exercise efficacy compared with other conditions, but this does not occur among male participants (Barha et al., 2017). Moreover, initial exploration via small-sample VR studies (Kocur et al., 2020) also showed that the Proteus effect on grip strength occurred only among male participants, indicating potential sex differences in the Proteus effect toward avatars with various muscularity levels. Therefore, sex is likely to moderate the association of avatar muscularity with exercise outcomes. However, very few studies have explored the role of sex on the Proteus effect in VR avatar embodiment (Kocur et al., 2020), and existing studies suffer from the use of a within-subject design and small sample sizes, which limit sex comparisons. Moreover, the direction of the moderation is unknown and should be explored. We thus propose the following research questions:

RQ1: How does sex moderate the relationship between avatar body shape and physical activity?
RQ2: How does sex moderate the relationship between avatar body shape and perceived exertion?
RQ3: How does sex moderate the relationship between avatar body shape and (a) immediate self-efficacy of core-muscle exercise and (b) next-day self-efficacy of core-muscle exercise?
RQ4: How does sex moderate the relationship between avatar body shape and altered self-concept?

Lastly, we positioned perceived body shape, body mass index (BMI), and BOI as covariates of all the analyses to control each participant’s perception of their own body shape, their BMI, and the degree they perceive that they embody the virtual body (i.e., BOI) (Maselli and Slater, 2013).

## Materials and Methods

To test the hypotheses, we employed a 2 (muscular body with a six pack vs. normal body) × 2 (male vs. female) between-subject factorial design experiment. Recruitment invitations were issued through the daily campus announcement email of a national university in northern Taiwan. Students who signed up for this study were asked to complete a screening questionnaire on their daily exercise behaviors, height, body weight, and perceived body shape (1 as not muscular at all and 7 as very muscular). Those who did not perceive their own body as muscular (under 5 points) were invited to the laboratory to participate in the experiment. A total of 96 participants (51 females) participated in this study and were randomly assigned to one of four conditions. The participants embodied a same-sex muscular body with a six pack or a normal body according to the condition to which they were assigned and exercised following a workout video in a virtual gym.

### Stimulus

A virtual scene depicting a gym with a mirror wall with a television screen hung on the upper left side of the mirror wall was created using the Unity engine^1^. The participants viewed the virtual world using an HTC VIVE head-mounted display (HMD). The participants’ actual bodies were replaced with a same-sex virtual body in the virtual scene. They could see their virtual body from the first-person point of view or in the mirror in front of them. We employed a Microsoft Xbox One Kinect Sensor to track the participants’ actual body postures and movements and mapped those movements onto the virtual body so they could see virtual body movements in accordance with their actual body movements in real time.

**FIGURE 1:**
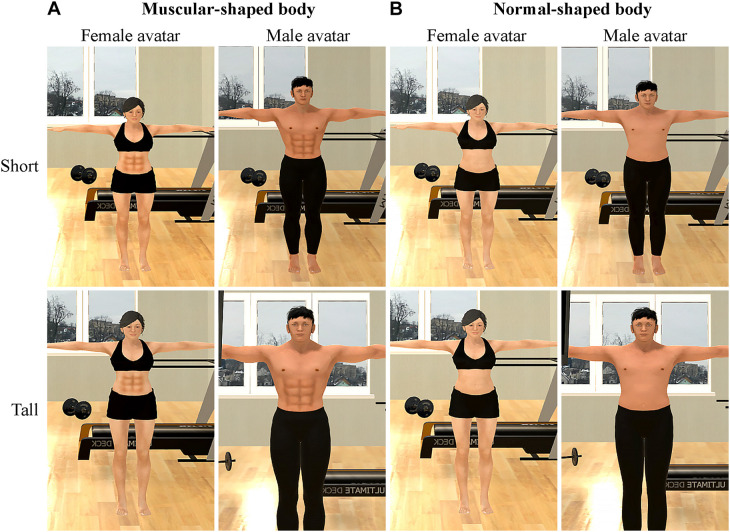
Avatars. **(A)** The female and male avatars with muscular-shaped bodies. **(B)** The female and male avatars with normal-shaped bodies. (The upper row shows the short avatars for the participants who were shorter, and the lower row shows the tall avatars for the participants who were taller).

The avatars were created using MakeHuman^2^. The muscular-shaped body avatars had obvious abdominal muscles (i.e., six pack), while the normal-shaped body avatars did not. To prevent the unexpected effects caused by height differences between the virtual body and the real body, eight avatars were created–male/female avatar with a tall and muscular body, male/female avatar with a short muscular body, male/female avatar with a tall normal body, and male/female avatar with a short normal body (Figure 1) – and assigned to participants according to their actual height. Male participants who were taller than 5′9′′ feet and female participants who were taller than 5′5′′ feet were assigned tall avatars, and other participants were assigned short avatars.

**FIGURE 2:**
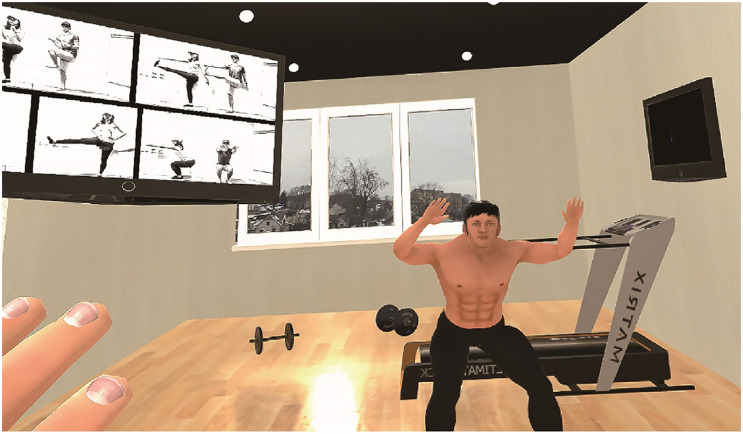
The environment of the virtual gym. The participants could see their virtual body from a first-person perspective and in the mirror.

The scenario began with the participant standing in a gym facing the mirror wall with a television screen on the mirror wall playing a core workout video. The participants were asked to observe and move their virtual body first and then exercise following the video on the screen (Figure 2).

### Procedure

**FIGURE 3:**
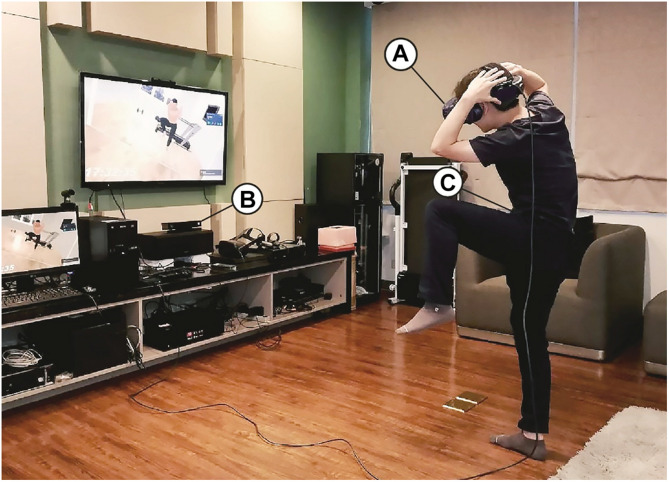
Experimental setup. **(A)** HTC VIVE head-mounted display (HMD). **(B)** Microsoft Xbox One Kinect Sensor for actual body movements tracking. **(C)** ActiGraph GT3X accelerometer for recording physical activity.

On arriving at the laboratory, the participants signed an informed consent form. Then, research staff directed the participants to a cleared floor space (approximately 3 × 2.5 m) and helped them put an ActiGraph GT3X accelerometer unit on their waist to record continuous physical activity. Afterward, research staff assisted the participants in wearing the HTC VIVE HMD and informed the participants that they would have a VR experience. The participants were asked to follow the prerecorded voice instructions in the virtual scene (Figure 3).

**FIGURE 4:**
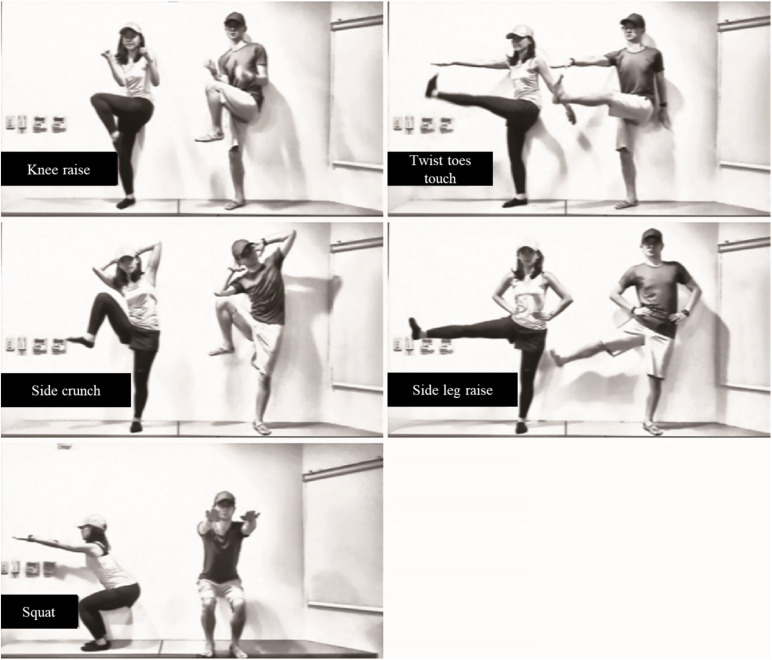
The standing core workouts in the video.

At the beginning of the VR experience, the participants found themselves in a virtual gym facing a large mirror wall. The voice instructions guided them to look around and observe their virtual bodies by looking down and looking in the mirror. Then, they were instructed to perform a series of movements, such as knee lifts and arm waves, to help them associate their physical body movements with the avatars’ movements. This phase lasted 40 s. Next, a video featured a series of standing core workouts played on the TV screen, and the participants were instructed to exercise following the video (Figure 4). The workout video lasted for 3 min and 48 s, followed by a free practice session for 2.5 min. The participants were told that they could keep exercising, practice the workouts in the video freely or hang out in the virtual gym until the time was up.

After that, the research staff helped the participants remove the HMD and accelerometer and asked the participants to rate their perceived exertion immediately. Then, the participants were guided to sit in front of the computer and complete the IAT and the rest of the study measures. A follow-up questionnaire link was sent out 24 h after the participant left the laboratory and asked about their self-efficacy for exercise.

### Measurements

#### Perceived Body Shape

Perceived body shape (*M* = 1.74, *SD* = 1.03) was assessed using a one-item scale developed by this study. The participants rated their body shape on a scale that depicted male/female normal-shape bodies without abdominal muscles at the left end (1 as not muscular at all) and male/female muscular bodies with six-pack muscles at the right end (7 as very muscular).

#### Perceived Exertion

Perceived exertion (*M* = 12.16, *SD* = 2.00) was assessed using the Borg Rating of Perceived Exertion (RPE; Borg, 1998) scale. The scale is a simple numerical list ranging from 6 (no exertion at all) to 20 (maximal exertion). The participants rated their exertion during the activity in the VR experience on the scale, combining all sensations and feelings of their physical stress and fatigue.

#### Self-Efficacy for Exercise

Self-efficacy for exercise (same day: α = 0.84, *M* = 4.26, *SD* = 1.31; next-day: α = 0.84, *M* = 4.09, *SD* = 1.19) was measured using a four-item scale modified from Lorig et al. (1996). The participants rated the degree of agreement (1 = strongly disagree and 7 = strongly agree) with statements such as “After the VR experience, I am confident that I can do some simple core workouts in my daily life” and “In the next 6 months, I am confident that I can do some core workout regularly.”

#### Automatic Self-Concept

Automatic self-concept was assessed using the IAT, which was developed to measure beliefs that people may be unable to report by detecting the strength of associations between concepts with a particular attribute (Greenwald et al., 1998). This test has been successfully used for the assessment of self-concept (Schnabel et al., 2008; Suslow et al., 2014). The participants were asked to quickly sort words related to 2 target concepts (self and others) and 2 attributes (normal body shape and muscular body shape) into categories shown on the left or right sides on the computer screen by pressing the P or Q key. The IAT score (*M* = 17.58, *SD* = 215.60) is calculated based on the participants’ response times. If participants perceived their figures as more muscular, they performed faster when hitting the response key for highly associated categories (e.g., self + muscular) than for less associated categories (e.g., self + normal figure).

We conducted the IAT using E-prime^3^ and following the procedure suggested by Greenwald et al. (1998). The test involved 5 sessions of discrimination tasks. The first two sessions were target-concept and attribute discrimination tasks. The participants practiced sorting words into “self” and “others” categories on the screen in the first session and into “muscular body shape” and “normal body shape” categories in the second session. The third session was a combined task, and the participants sort the words into two combined categories, each including one target and one attribute concept and using the same key in the preceding two steps. After that, the participants practiced another target-concept discrimination task with reversed key assignments in the fourth session. Then came a reversed combined task using the same response key assignments as the second and fourth sessions. The words used for the discrimination tasks are shown in Table 1. The orders of Session 2–3 and Session 4–5 were counterbalanced. The IAT score was obtained from the difference in mean response times between the two combined tasks with reversed combinations of concepts (i.e., Session 3 and Session 5). Before starting the calculation, those trials with a response time greater than 3,000 ms were recoded to 3,000 ms, and those that were less than 300 ms were recoded to 300 ms. The participants with error rates higher than 20% were dropped from the IAT analysis. The IAT score was interpreted in accordance with the notion that the more negative (or less positive) a person’s IAT score is, the greater their association of normal body shape with the self; on the other hand, the more positive (or less negative) a person’s IAT score is, the greater their association of muscular body shape with the self.

**TABLE 1:** The words used for the Implicit Association Test (IAT).

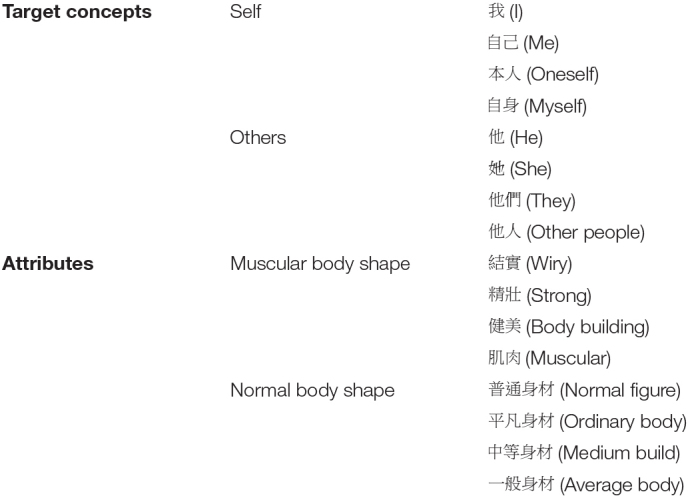

*The test was conducted in Chinese, so we listed the original Chinese words used in the test and tried to translate them into English words with similar meanings.*

#### Physical Activity

Physical activity was collected using the ActiGraph GT3X accelerometer and ActiLife software, as these are established measurements (Dyrstad et al., 2014). The sampling rate was set to 100 per second, and the epoch was set to 10 s, which means that it integrated 1,000 samples every 10 s to produce one data point. The filtering and exporting process was performed using ActiLife software. The exported motion data were vector magnitudes (*M* = 5274.45, *SD* = 2181.69), that is, the total amount of movements on the three axes, which is calculated from the square root of the sum of the squares of all three axes.”

#### Body Ownership Illusion

Body ownership illusion (α = 0.74, *M* = 4.2, *SD* = 0.94) was measured using an 8-item scale modified from Hasler et al. (2017) and Grechuta et al. (2019)The items included “Although the virtual body did not look like me, I had the sensation that the virtual body I saw in the mirror was mine,” “Although the virtual body did not look like me, when looking down at my body I had the sensation that it was mine,” and “When I looked down, it seemed as if I had more than one body (reverse coded item).”

## Results

To examine the hypothesis and research questions, we employed 2 (avatar body shape: muscular with a six pack vs. normal) × 2 (sex: male vs. female) between-subject ANCOVA analyses on physical activity, self-efficacy, RPE and IAT reaction time data, with participants’ perceived body shape, BMI, and BOI in VR as covariate variables. Among all 96 participants, 25 males and 23 females were assigned to the muscular avatar with a six pack group, and 20 males and 28 females were assigned to the normal avatar group. The average age of our sample was 21.56, ranging from 18 to 42 years, and an independent *t*-test showed no significant difference in the mean age between the two groups, *t*(94) = 0.42, *p* > 0.05.

H1 and RQ1 concern the relationship between avatar body shape and users’ physical activities among male and female participants. An ANCOVA analysis of accelerometer data showed that during the follow-along workout session, there was a significant group main effect on physical activity [vector magnitude: *F*(1,89) = 5.77, *p* < 0.05, η^2^ = 0.06], the participants in the normal avatar group made more movements (adjusted *M* = 5748.67, *SD* = 285.53) than those in the muscular avatar with a six pack group (Adjusted *M* = 4761.97, *SD* = 284.50). Although no other significant effects were found during the follow-along workout session or free practice session, these results did provide some evidence for H1; players with a normal avatar body shape displayed more physical activity than those with a muscular body shape with a six pack.

The participants also reported the RPE scale to estimate their subjective physical exertion. An ANCOVA analysis of the RPE score was conducted to test H2, which anticipates RPE differences between the normal avatar group and the muscular avatar with a six pack group. Nevertheless, neither main effects of avatar body shape [*F*(1,89) = 0.53, *p* > 0.05, η^2^ = 0.01] and sex [*F*(1,89) = 0.02, *p* > 0.05, η^2^ < 0.01] nor interaction [*F*(1,89) = 0.11, *p* > 0.05, η^2^ < 0.01] were significant on the participants’ RPE scores. Thus, H2 was not supported, as different avatar body shapes did not affect the players’ perceived exertion.

**FIGURE 5:**
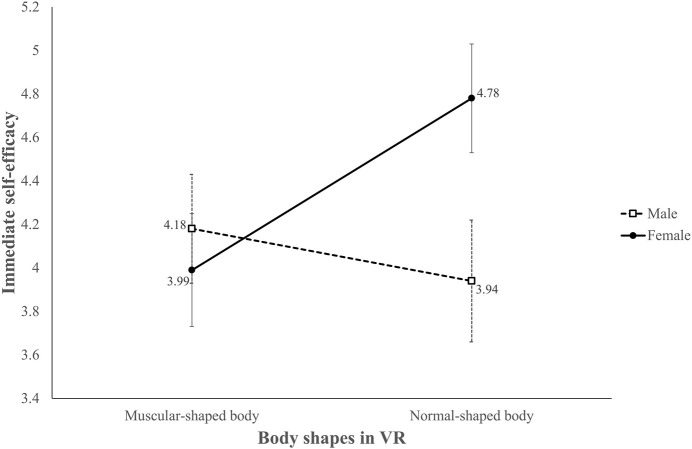
Adjusted mean score of immediate self-efficacy for core workouts.

Regarding immediate self-efficacy for core-muscle exercise (H3a and RQ3a), the ANCOVA showed a nearly significant interaction between avatar body shape and sex, *F*(1,89) = 3.87, *p* = 0.052, η^2^ = 0.04 (Figure 5). We further conducted a *post hoc* analysis and found that the effect of avatar body shape existed only in females, *F*(1,89) = 5.22, *p* < 0.05, η_*p*_^2^ = 0.06; those who embodied normal avatars demonstrated higher self-efficacy (adjusted *M* = 4.78, *SE* = 0.25) than their muscular avatar with a six pack counterparts (adjusted *M* = 3.99, *SE* = 0.26). We also found a significant sex difference in the normal avatar group, *F*(1,89) = 5.50, *p* < 0.05, η_*p*_^2^ = 0.06, and females showed higher self-efficacy (adjusted *M* = 4.78, *SE* = 0.25) than males (adjusted *M* = 3.94, *SE* = 0.28).

**FIGURE 6:**
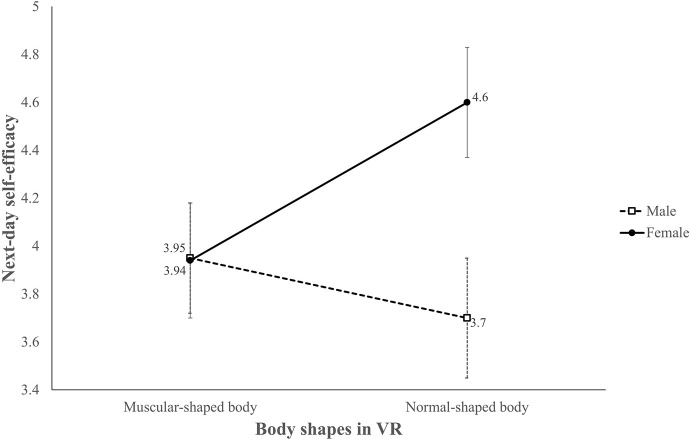
Adjusted mean score of next-day self-efficacy for core workouts.

Similar results were found in next-day self-efficacy (H3b and RQ3b). Although the interaction between avatar body and sex was not significant [*F*(1,89) = 3.71, *p* = 0.057, η^2^ = 0.04] we still observed a simple main effect of avatar body shape in females [*F*(1,89) = 4.49, *p* < 0.05, η_*p*_^2^ = 0.05] and a sex difference in the normal avatar group [*F*(1,89) = 7.70, *p* < 0.01, η_*p*_^2^ = 0.08]. Similar to immediate self-efficacy, the female participants in the normal avatar group demonstrated higher next-day self-efficacy (adjusted *M* = 4.60, *SE* = 0.23) than the female participants in the muscular avatar with a six pack group (adjusted *M* = 3.94, *SE* = 0.24) and the male participants in the normal avatar group (adjusted *M* = 3.70, *SE* = 0.25; Figure 6). We also conducted a three-way mix designed ANCOVA (with avatar body shape and sex as between subject variables and measure date as within-subject variable) to probe the change of self-efficacy in 2 days; the results showed neither a main effect [*F*(1,89) = 1.46, *p* > 0.10, η_*p*_^2^ = 0.02] nor any interaction of measure date (all *F*s < 1, *p*s > 0.10), suggesting that participants’ self-efficacy did not change during two measured time regardless of avatar body shape and sex. According to these results, participants who embodied a normal body shape avatar will display more self-efficacy for core-muscle exercise, yet this effect appears only among female users.

To understand the influence of avatar body shape on users’ automatic self-concept (H4) and the potential moderating effect of sex (RQ4), we used the IAT to probe the participants’ automatic self-concepts. Following the procedure (Greenwald et al., 1998), the IAT score was derived by subtracting the average reaction time (RT) of session 3 (other-normal and self-muscular combination) from the average RT of session 5 (other-muscular and self-normal combination). A negative score indicates that the linkage between self and normal was stronger than the linkage between others and normal, and a positive IAT score indicates the opposite relation.

**FIGURE 7:**
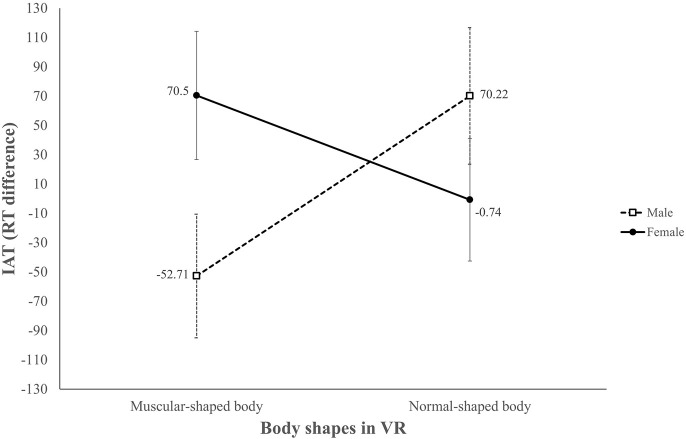
Interaction between avatar body shape and sex of participants in predicting participants’ self-concept after the VR experience.

A two-way interaction was found in the ANCOVA analysis, *F*(1,89) = 4.93, *p* < 0.05, η^2^ = 0.05. Automatic self-concept differed males and females in the muscular avatar with a six pack group, *F*(1,89) = 4.23, *p* < 0.05, η^2^ = 0.05, while there was no significant difference in the normal avatar group (Figure 7). In the muscular avatar with a six pack group, males connected the concept of “normal body” with “self” more strongly than with “others,” yet females showed a reverse pattern: they connected “normal body” with “others” more strongly than with “self”. No main effects were found. In other words, a muscular avatar with a six pack did not trigger the participants’ positive self-concept, and H4 was not supported. However, the effect of avatar body shape on the participants’ self-concepts was moderated by sex, with female participants associating themselves more with the muscular avatar with a six pack and the male participants associating themselves more with the normal-body avatar (RQ4).

## Discussion

This study aimed to investigate the Proteus effect from a first-person perspective and during avatar embodiment in actual exercise. In addition to the immediate measurements of the Proteus effect, prolonged effects such as next-day perception and exercise-related outcomes were also explored. We also theorized the Proteus effect as occurring due to an altered perceived self-concept and explored the association between VR avatar manipulation and self-concept in the exercise context. While the existing literature has mainly investigated the Proteus effect in a non-VR environment or explored the effect after VR embodiment, we aimed to contribute to the literature by addressing this concern by exploring how the Proteus effect works in actual VR exercise. In addition, we explored the role of sex as a potential moderator in the association of the Proteus effect on exercise outcomes. Furthermore, the between-subject design of the study allowed us to investigate how avatar manipulation of muscular body shape with a six pack as opposed to normal body shape influenced participants’ self-concept and exercise outcomes, as limited VR studies have employed within-subject comparisons. The findings also contribute to the literature by providing an upward comparison (e.g., muscular vs. normal) as opposed to the previous downward comparison regarding body fitness (e.g., normal vs. obese).

Through a 2 (avatar body shape: muscular vs. normal) × 2 (sex: male vs. female) between-subject experiment, the results partially support the Proteus effect. The participants embodying a normal-body avatar demonstrated more physical activity than those with a muscular avatar when performing core-muscle exercise during avatar embodiment. This may support our hypothesized Proteus effect that embodying a muscular avatar with a six pack results in a firmer position during exercise and limits extraneous body movements. However, physical activity might also reflect that one performs the exercise less effectively. In other words, participants who embodied the avatar with the six pack might perceive themselves as not needing to exercise that much, resulting in fewer physical movements (e.g., underperformance). Not knowing the exact rationale and psychological processes, we can only provide our observation that most participants followed the instructions in the video to engage in the exercise seriously during the teaching time (which is the main analysis conducted) and underperformance should not be an issue. Nevertheless, more detailed effects on their physical activity should be probed in future research.

Avatar manipulation of body shape in VR did not affect the participants’ perceived exertion during the core-muscle training exercise. This conflicts with the findings of Kocur et al. (2020), in which participants perceived lower exertion when embodying a muscular avatar. Two reasons may explain the lack of effect found in this study. First, the participants engaged in several core-muscle training exercises in our study, and thus, they paid more attention to following the exercise movements during the embodiment. Kocur et al. (2020) allowed participants to lift weights or flex their muscles while looking at their virtual body, which may highlight the importance of avatar body shape. Another reason may be that these exercises do not provide much variance in exertion. The participants may not have perceived the exercises as difficult. More research is needed to further examine this issue.

The Proteus effect occurs in not only the physical dimension (i.e., physical activity) but also the psychological dimension (i.e., self-efficacy). Regarding immediate self-efficacy, the Proteus effect was found among female participants but not male participants. The immediate the next-day self-efficacy for core-muscle exercise had the same pattern, with female participants who embodied normal avatars reporting greater self-efficacy. This suggested that the female participants who embodied the normal avatars might have perceived the avatar body shape as closer to their actual body and as needing more exercise to improve it, and the workout following the video session in VR increased their knowledge and confidence for pursuing this goal, resulting in greater self-efficacy. On the contrary, the female participants who embodied muscular with a six pack avatars might have perceived their bodies as good enough and therefore lack sufficient motivation to exercise more. These results also indicated that the Proteus effect occurs not only immediately after avatar embodiment but lasts until the next-day. Whereas Reinhard et al. (2020) reported the Proteus effect after avatar embodiment, we further showed that it lasts until the next-day. This is important for the theory of the Proteus effect because the underlying mechanism is key for the outcomes and important for practitioners to efficiently boost exercise motivation through VR muscular avatar embodiment.

Self-concept was explored in this experiment, and it was an important outcome of the Proteus effect. However, the impact of the effect on self-concept was only partially supported because the outcomes varied by sex: the Proteus effect, as altered self-concept, was found among females but not males. In addition, whereas the female participants associated the muscular with a six pack concept with themselves more than with other people, the male participants perceived their self as more normal than other people. This showed that the upward comparison of avatar body shape or more ideal body may have backfired for the male participants. As having a six pack (Hoyt and Kogan, 2001; Drummond and Drummond, 2014; McNeill and Firman, 2014) was heavily emphasized as an attribute of an ideal and muscular body, it may have triggered the male participants’ body stereotypes and thus increased awareness or reactance to this persuasion tactic. In other words, the “obvious” persuasive cue may remind them of their real body shape without a six pack. Another explanation is that people can feel their core muscles, but merely seeing the embodied avatar having a six pack without feeling those muscles flex may serve as an inconsistency between the virtual and actual body, which may break the Proteus effect and trigger a sense of disbelief (Banks et al., 2019). As a sense of disbelief dampens the player-avatar relationship (Banks et al., 2019), the inconsistency requires further investigation.

The results also showed that sex is an important moderator of the VR Proteus effect, specifically in the exercise context. The female and male participants showed distinctive patterns and even opposite perceptions of self-concept and exercise outcomes. Existing studies have examined the Proteus effect among only male or female participants (e.g., Fox et al., 2013) or suffer from a sample size for both sexes that was insufficient for comparing the effects (e.g., Yee et al., 2009) in the VR embodiment context. Our study is the first to examine the role of sex differences in VR embodiment from the first-person perspective during actual exercise. We found that consistent with an argument in the literature (e.g., Hargreaves and Tiggemann, 2003; Levine and Murnen, 2009), the female participants internalized idealized body image more than the male participants. More precisely, although current Taiwanese society positions muscular body shape with a six pack as ideal, we further found that the female participants internalized muscular body shape more than the male participants, regardless of their preferred ideal body shape. Our results showed that the female participants who embodied the muscular avatar perceived themselves as more muscular than others. The muscular body shape with a six pack served as a cue for the female participants in the exercise context but served as a cue that may have triggered reactance or dissonance among the male participants, thus leading to the counter effect of perceiving themselves as having a more normal body shape than others. Simply put, sex differences exist in motivation outcomes such as self-efficacy and perceived self-concept, as the muscular avatar shape resulted in opposite effects for the female and male participants. Future research should investigate sex differences in the Proteus effect and the underlying mechanisms in various contexts.

The physical movement results of the accelerometer are the only significant evidence that supported the Proteus effect in this study. The same pattern with only partial support also occurred in another study exploring participants’ walking speed after avatar embodiment (Reinhard et al., 2020). These results indicate that the Proteus effect may have complicated underlying psychological processes, especially in the exercise context, and thus providing partial positive evidence of the effect. More research is needed.

When interpreting the above results, several limitations exist. First, the duration of avatar embodiment for exercise during the follow-along and the free practice session was short, less than 10 min. In addition, the series of movements for the core-muscle exercise was moderate-difficult, so these movements may not provide much variance in perceived exertion. Future research should replicate this study and choose the more intense movements that require more effort to examine the Proteus effect on perceived exertion as an exercise outcome. Second, some participants did not know what to do in the practice session, whereas other participants engaged in rigorous training, such as pushups or planks. The participants’ knowledge of or habits for core-muscle training are potential covariates for future research. In addition, the necessity of including a practice session in the experimental design requires more discussion and empirical evidence. Third, we did not measure the individual’s ideal body shape as a control variable in our study. As each person may have a different preferred body shape, a virtual body with a six pack may not represent the ideal body shape for the participants. Nevertheless, thanks to the nature of the random assignment in the experimental method, individual differences should be equally distributed across conditions. We do not take this as an excuse; rather, we suggest that future research should determine participants’ ideal body shape to further examine underlying psychological processes. Fourth, with our limited technique in creating the virtual avatars, we were able to create only tall and short avatars for this research. Dynamical scaling of the participant’s height or 3D body scans (Thaler et al., 2018; Pujades et al., 2019) are recommended to create virtual avatars for future research. Last, we employed the motion tracking system through a Kinect camera to mirror the participants’ physical movements so that they could freely move their bodies without having to wear sensors on their knees, ankles, and wrists. Although we have done our best to ensure this freedom, some participants indicated that the VR experience was not smooth because the avatar did not always reflect their detailed movements without delay. We have addressed this issue by including the BOI as a covariate in our analysis. We still recommend that future research employ tracking suits or advanced tracking systems to examine the Proteus effect in VR exercise.

## Conclusion

We contributed to the literature by examining the Proteus effect in first-person perspective avatar embodiment in the context of exercise. In addition, the participants engaged in actual aerobic exercise as opposed to merely walking or lifting weights. Both immediate and prolonged effects were explored in this study to assess the potential next-day presence of the Proteus effect, and we found a prolonged effect only for self-efficacy and only among female participants. We further demonstrated that sex differences were important factors to consider in research on the Proteus effect in the exercise context. Specifically, the female participants demonstrated more positive exercise motivation after embodying normal avatars than after embodying muscular avatars. Previous studies examining the effects of an avatar’s body shape on healthy intentions and behaviors mainly focused on comparing a normal body against an obese body and indicated that compared to the obese avatar, using a more ideal-shaped avatar has more positive outcomes (Li et al., 2014; Peña et al., 2016; Joo and Kim, 2017). This study adds new insights to the literature by providing an upward comparison of the avatar’s body shape (i.e., normal vs. muscular with an emphasis of a six pack) and found that the effects of embodied exercise in VR did not yield better effects for the more ideal figure embodied. Compared to the muscular-shaped avatar, the normal-shaped avatar has more positive effects on self-efficacy for exercise among female participants. Moreover, the female participants who embodied the muscular avatars demonstrated a more positive self-concept than those who embodied the normal avatars. This result suggested that the Proteus effect was present when using a muscular avatar with a six pack and altered the female participants’ self-concept and boosted their body image; however, it may also have hindered their motivation for future exercise participation. These factors need to be considered in the design of sports-related experiences in the future. Further, the muscular avatar shape resulted had opposite effects on altering self-concept among male participants, indicating that the Proteus effect was effective among the female participants when using muscular body shapes for persuasion, but such persuasion was not present for the male participants. Persuasive mechanisms and reactance toward the body-shape-related Proteus effect may vary by sex. Future research should examine these important sex variations and differences in exercise outcomes.

## Data Availability Statement

The raw data supporting the conclusions of this article will be made available by the authors, without undue reservation.

## Ethics Statement

The studies involving human participants were reviewed and approved by the National Chengchi University IRB. The patients/participants provided their written informed consent to participate in this study. The individual(s) provided their written informed consent for the publication of any identifiable images or data included in this article.

## Author Contributions

J-HL designed the study and wrote the entire manuscript (except for the method and results section). D-YW executed the entire experiment and assisted with partial manuscript drafting. J-WY assisted in analyzing the data and wrote the results section. All authors contributed to the article and approved the submitted version.

## Conflict of Interest

The authors declare that the research was conducted in the absence of any commercial or financial relationships that could be construed as a potential conflict of interest.

## Publisher’s Note

All claims expressed in this article are solely those of the authors and do not necessarily represent those of their affiliated organizations, or those of the publisher, the editors and the reviewers. Any product that may be evaluated in this article, or claim that may be made by its manufacturer, is not guaranteed or endorsed by the publisher.

## Footnotes

1
https://unity.com/

2
http://www.makehumancommunity.org/

3
https://pstnet.com/products/e-prime/
