# Corrigendum: Exercising With a Six Pack in Virtual Reality: Examining the Proteus Effect of Avatar Body Shape and Sex on Self-Efficacy for Core-Muscle Exercise, Self-Concept of Body Shape, and Actual Physical Activity

**DOI:** 10.3389/fpsyg.2022.857059

**Published:** 2022-03-09

**Authors:** Jih-Hsuan Tammy Lin, Dai-Yun Wu, Ji-Wei Yang

**Affiliations:** ^1^Department of Advertising, College of Communication, National Chengchi University, Taipei City, Taiwan; ^2^Taiwan Institute for Governance and Communication Research, Taipei City, Taiwan; ^3^Department of Communication and Technology, National Yang Ming Chiao Tung University, Zhubei, Taiwan

**Keywords:** avatar body shape, self-concept, self-efficacy, female and male, Proteus effect, virtual reality, virtual reality exercise, physical activity

In the original article, there was a mistake in Figure 5, Figure 6, and Figure 7 as published. The labels of the horizontal axis were incorrectly typed as “bpdy shapes in VR.” This should be corrected as “body shapes in VR.” The corrected Figure 5, Figure 6, and Figure 7 appear below.

**Figure 5:**
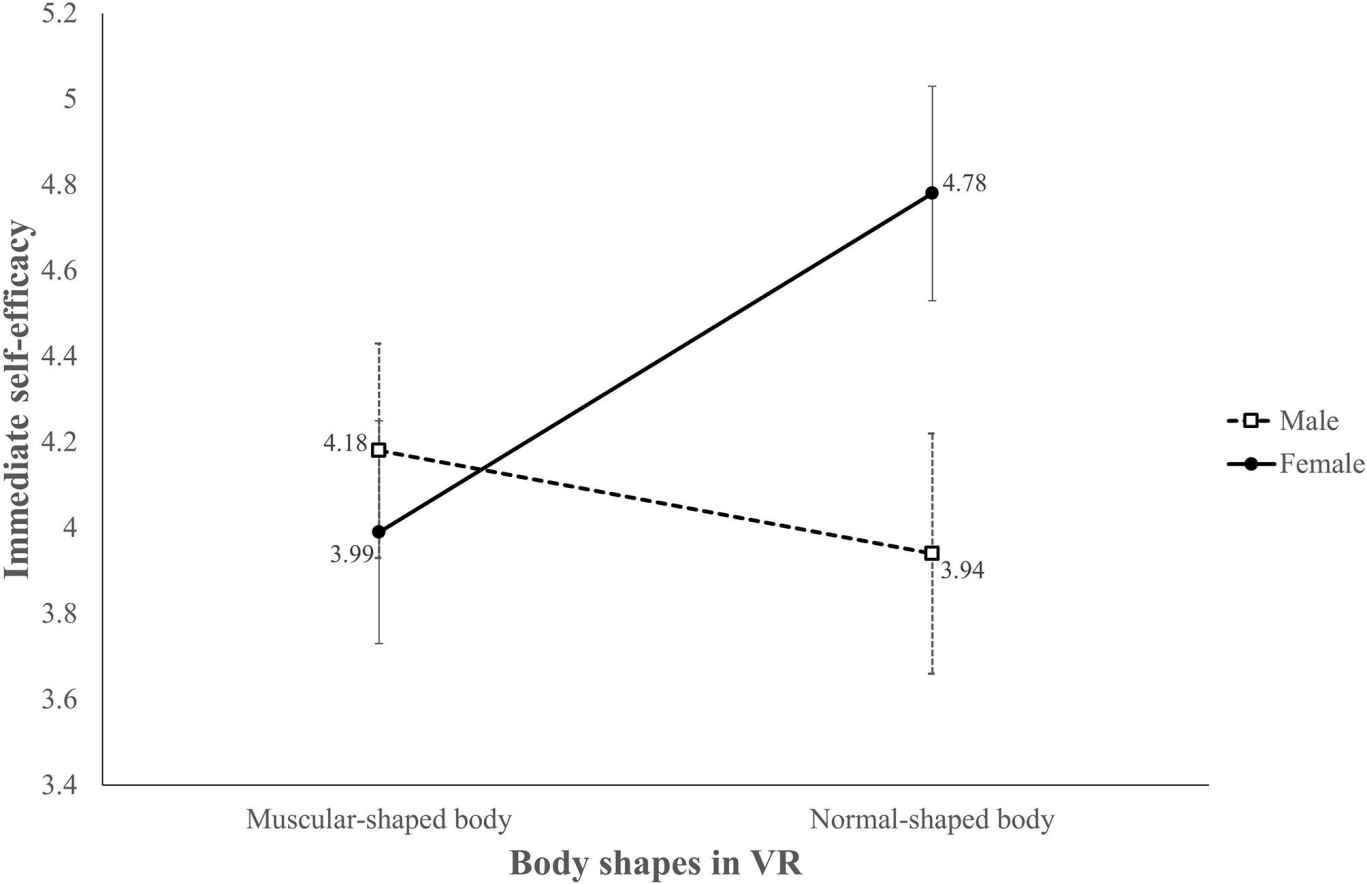
Adjusted mean score of immediate self-efficacy for core workouts.

**Figure 6:**
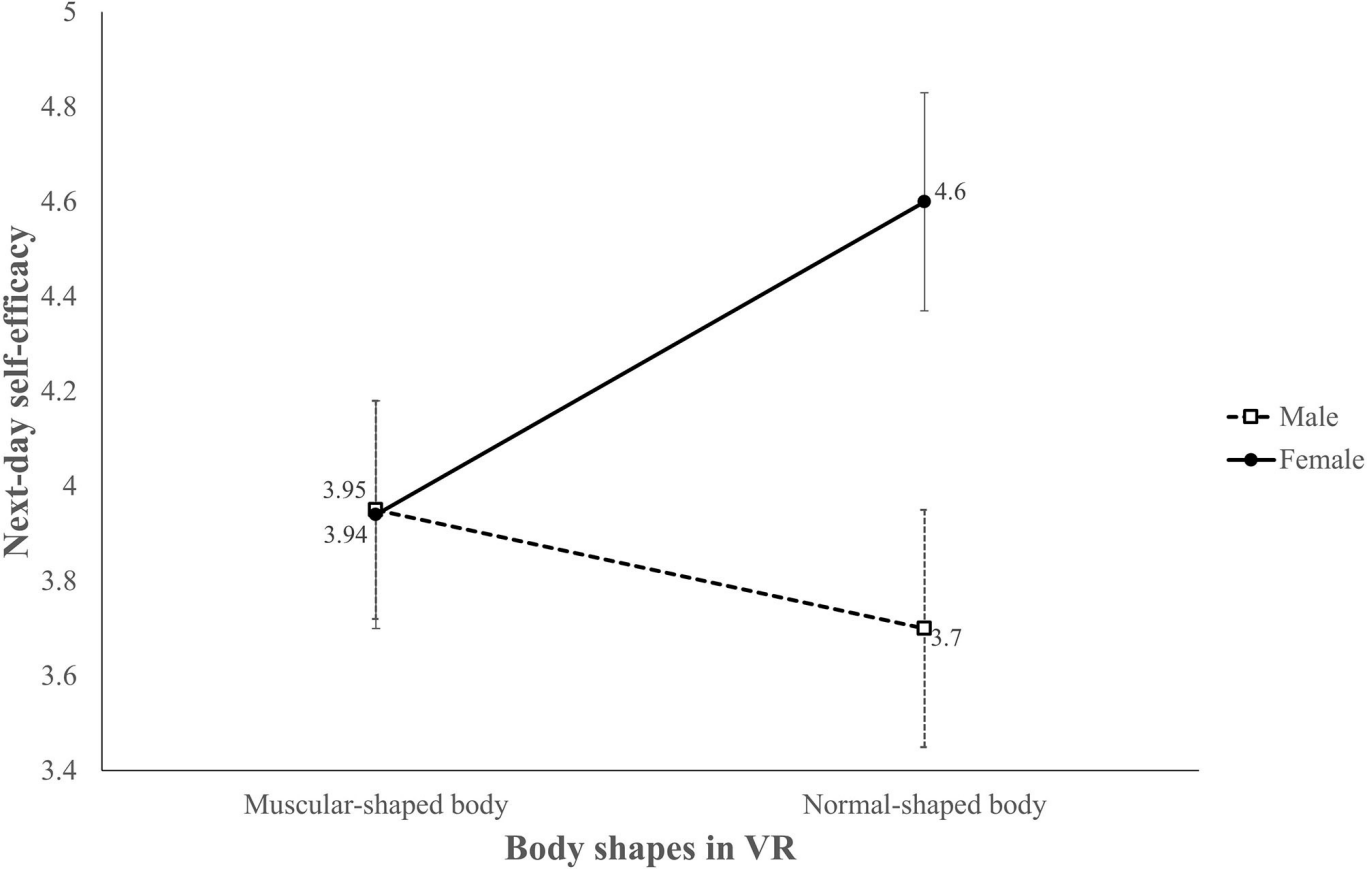
Adjusted mean score of next-day self-efficacy for core workouts.

**Figure 7:**
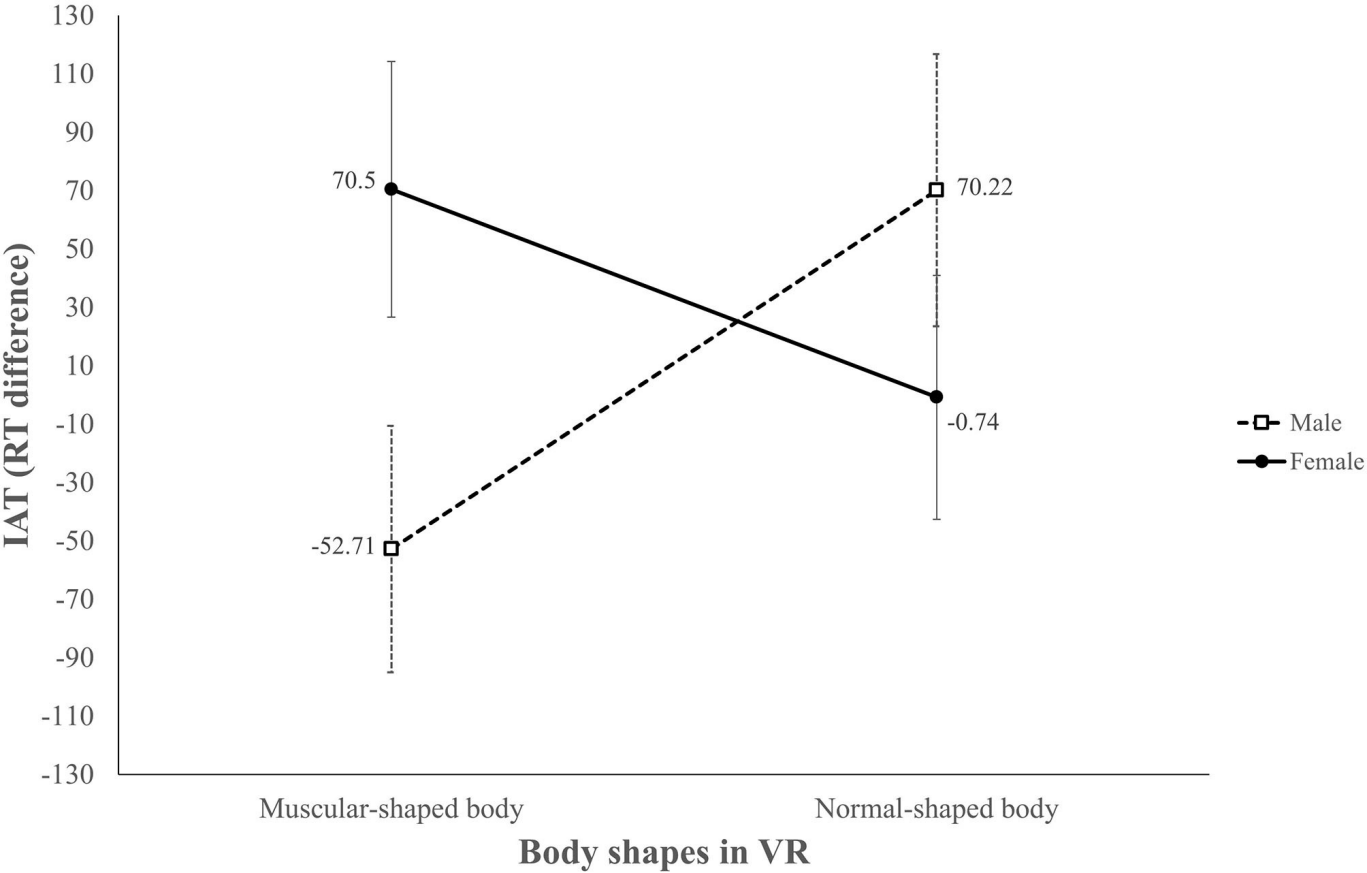
Interaction between avatar body shape and sex of participants in predicting participants' self-concept after the VR experience.

The authors apologize for this error and state that this does not change the scientific conclusions of the article in any way. The original article has been updated.

## Publisher's Note

All claims expressed in this article are solely those of the authors and do not necessarily represent those of their affiliated organizations, or those of the publisher, the editors and the reviewers. Any product that may be evaluated in this article, or claim that may be made by its manufacturer, is not guaranteed or endorsed by the publisher.

